# Editorial: Ion Channels: Therapeutic Targets for Neurological Disease

**DOI:** 10.3389/fnmol.2021.797327

**Published:** 2021-11-16

**Authors:** Panpan Hou, Xiaona Du, Hailong An

**Affiliations:** ^1^Dr. Neher's Biophysics Laboratory for Innovative Drug Discovery, Macau University of Science and Technology, Taipa, Macao SAR, China; ^2^State Key Laboratory of Quality Research in Chinese Medicine, Macau University of Science and Technology, Taipa, Macao SAR, China; ^3^Department of Pharmacology, Hebei Medical University, Shijiazhuang, China; ^4^The Key Laboratory of Neural and Vascular Biology, Ministry of Education, Hebei Medical University, Shijiazhuang, China; ^5^The Key Laboratory of New Drug Pharmacology and Toxicology, Hebei Medical University, Shijiazhuang, China; ^6^Key Laboratory of Molecular Biophysics of Hebei, Institute of Biophysics, School of Science, Hebei University of Technology, Tianjin, China

**Keywords:** ion channels, drug target, neurological disease, single nucleotide polymorphism (SNP), hearing loss, spinal cord injury (SCI), schizophrenia, epilepsy

Contributing 11.6% of global DALYs (disability-adjusted life-years) and 16.5% of deaths from all causes, neurological disorders remain the leading cause of DALYs and the second leading cause of deaths (following cardiovascular diseases) over the world (Feigin et al., 2019). Neurological disorders are public health challenges that cause not only a decline in the quality of life for patients, but also a substantial burden for every family (Feigin et al., 2020). The prevalence of most neurological disorders increases with age, and the lack of effective treatment options continues to increase the number of patients (Feigin et al., 2019). Therefore, developing new strategies to prevent and treat the major neurological disorders is of great importance to improve human health.

Neurons communicate *via* rapid electrical activities that allow the nervous system to coordinate sensation, behavior, and emotion. Ion channels in neurons are the main information carrier of these neuronal electrical activities. They form conduction pores to allow selected ions (Na^+^, K^+^, Ca^2+^, Cl^−^, etc.) to pass through the cell membrane and generate electrical signals for establishing the resting membrane potential (Du et al., 2014), shaping each phase of action potentials, and controlling the Ca^2+^ signaling etc. (Hou et al., 2016; Chiamvimonvat et al., 2017; Shi et al., 2021). Dysfunction of ion channels by inherited mutations, pathological changes, or unwanted drug-induced side effects can alter ion flux across the membrane and cause neurological disorders. On the other hand, tremendous pre-clinical and clinical studies suggest that regulating the function of key ion channels involved in diseases can effectively alleviate the symptoms, supporting that ion channels are promising targets for treating neurological disorders.

In this topic named “*Ion Channels: Therapeutic Targets for Neurological Disease*,” we collect and summarize the most recent studies including four original articles and two reviews focusing on ion channel modulation mechanisms and ion channels as potential targets for treating hearing loss, pain, spinal cord injury, ischemia-reperfusion injury, and schizophrenia.

Single nucleotide polymorphisms (SNPs) refers to DNA sequence polymorphisms caused by mutation of a single nucleotide. It is the most common form of genetic variants in humans, accounting for more than 90% of all known polymorphisms (International HapMap et al., 2007). Studies indicate that SNPs are associated with the severity of illness, such as Alzheimer's disease (Burns et al., 2011). SNPs are also common in ion channels. Plante et al. examined the effects of several non-synonymous SNPs on the *KCNMA1* gene encoding the large conductance potassium (BK) channel (Atkinson et al., 1991). It turned out these SNPs can induce gain-of-function or loss-of-function effects on BK channels. For example, the SNP R800W slows down the activation rate, right shifts the conductance–voltage relation, and increases the amplitude of action potential-evoked currents. These loss-of-function effects are also conserved in the epilepsy-associated mutation D434G channel background. Overall, Plante et al. set a clear example that SNPs can modulate the function of BK channels. More basic and translational studies (for example the drug sensitivity change of SNPs) can be done to further understand the phenotypic and functional consequences of SNPs in ion channels.

The low-voltage activated (LVA) T-type calcium channel Cav3.2 sets a low threshold for action potential firing in dorsal root ganglion (DRG) neurons and plays a key role in pain sensation (White et al., 1989; Cain and Snutch, 2010). Ca_V_3.2 channel is regulated by different proteins (Zhang et al., 2013; Weiss and Zamponi, 2017), including the neuronal actin-binding protein Kelch-like 1 (KLHL1; Aromolaran et al., 2009, 2010). Using KLHL1 knockout (KO) or knock-down mice models, Martínez-Hernández et al. found that KLHL1 is important for maintaining the Ca_V_3.2 channel expression in DRG neurons. The Ca_V_3.2 protein level in KLHL1 KO mice is specifically decreased, and the T-type current is smaller than the wild type without significant change in the voltage-dependent activation. These results clearly demonstrate that by modulating the Ca_V_3.2 channel expression, KLHL1 can regulate the DRG excitability and pain sensitivity, providing a potential target to treat peripheral pain.

Unlike voltage-gated ion channels that sense the membrane voltage change *via* the voltage sensing domain (Wang et al., 2017; Hou et al., 2020), the transient receptor potential channels (TRP channels) are ligand-gated ion channels that are sensitive to physical (heat, pH, osmolarity, etc.) and chemical (ligands, Ca^2+^, etc.) stimuli from the environment, and therefore are important sensors for perception (Clapham, 2003). TRPV channels (the vanilloid subtype) are widely expressed and critical to nociception (Julius, 2013). TRPV3 is highly expressed in the cochlea hair cells, colocalizing with TRPV4 (Ishibashi et al., 2008). Studies on TRPV3KO mice model by Wang et al. found that a significant portion (27.7%) showed impaired hearing associated with loss of cochlear hear cells, but most TRPV3KO mice (72.3%) had normal hearing. Interestingly, compensatory upregulation of TRPV4 was observed in the TRPV3KO mice with normal hearing, suggesting that both TRPV4 and TRPV3 channels are important for maintaining normal hearing. Overall, these results offer not only novel insight into the molecular basis of hearing but also potential target for treating hearing loss.

The Ca^2+^ ion is also a key player for spinal cord injury (SCI), a seriously debilitating event that can lead to paralysis and even death (Ditunno and Formal, 1994). After SCI, damaged neurons release high concentrations of the neurotransmitter glutamate (Park et al., 2004), resulting in excessive intracellular Ca^2+^ and increasing cell death. To facilitate functional recovery after SCI, the combination of ion channel inhibitors to block the three main Ca^2+^ channels [Lom: for voltage-gated Ca^2+^ channels (Sattler et al., 1996), oxATP: for P2X_7_ receptors (Hollmann et al., 1991), and YM872: for Ca^2+^ permeable AMPA receptors (Hollmann et al., 1991)] has been proven an effective treatment for neurotrauma (O'Hare Doig et al., 2016, 2017). O'Hare Doig et al. further investigated this combinational strategy on a clinically relevant model of SCI and observed significant positive changes in early functional recovery and pathophysiology. Future studies on the combination of these ion channel blockers in chronic models of SCI are required to evaluate its long-term effect of treatment. Overall, O'Hare Doig et al. showed that the combination of Ca^2+^ channel blockers can be a promising strategy for treating SCI.

Cerebral ischemia-reperfusion injury (CIRI) can cause severe damage to the brain. During cerebral ischemia-reperfusion, oxidative stress occurs and a large number of inflammatory cytokines are present in the ischemic focus (Lin et al., 2016). Wu et al. summarized current studies about the roles of oxidative stress and inflammation in CIRI, as well as important signaling pathways and therapeutic options for CIRI. Similar to the abovementioned combination strategy works better for SCI than using individual blockers (Savigni et al., 2013), single antioxidant therapy can only reduce cerebral ischemic injury to a certain extent, and so far there is no simple strategy to control the neuroinflammation due to the complex mechanisms of inflammation in CIRI. To this point, Wu et al. proposed that future studies can focus on the development of a free radical scavenger with multiple mechanisms of action and the combination of free radical scavengers with anti-inflammatory drugs.

Schizophrenia is a serious mental illness that affects ~1% of the world population and places a major socio-economic burden (Blot et al., 2013). The World Health Organization (WHO) estimated that, in Western countries, direct annual costs of schizophrenia range from 1.6 to 2.6% of total health care expenditures. In the US alone, the annual economic burden of schizophrenia is more than US$60 billion (Chong et al., 2016). The N-methyl-D-aspartate receptor (NMDAR) is important for the development of the nervous system and the formation of neuronal circuits (Moghaddam et al., 1997; Fellin et al., 2009), and accumulating evidence in human and animal studies support that NMDAR hypofunction is a convergence point of various symptoms of schizophrenia. Lee and Zhou summarized various animal models of NMDAR hypofunction generated by both pharmacological and genetic approaches, and how they relate to the pathophysiology of schizophrenia. Lee and Zhou also discussed limitations of these animal models and their potential utility for therapeutic applications. With the recent breakthrough on structural and pharmacological studies of NMDAR (Zhang et al., 2021), these animal models will provide useful platforms to identify novel therapeutics for schizophrenia.

Taken together, neurological disorder is one of the leading cause of deaths all over the world. This research topic highlights exciting new advances in the modulation mechanism of key ion channels in the nervous system, and the critical roles of ion channels in maintaining the physiological function and alleviating the symptoms of diseases. Further basic and clinical studies on ion channels will help develop new therapeutics for treating neurological disorders and improving human health.

## Author Contributions

All authors contributed equally to the writing and editing of the manuscript.

## Funding

This work was supported by the National Natural Science Foundation of China (Nos. 32171221 to PH, 81870872 to XD, and 81830061 to HA), the Natural Science Foundation of Tianjin of China (No. 19JCYBJC28300 to HA), and the Natural Science Foundation of Hebei Province of China (No. H2020202005 to HA). This work was also funded by Dr. Neher's Biophysics Laboratory for Innovative Drug Discovery (Grant no. 001/2020/ALC) as supported by the Macau Science and Technology Development Fund.

## Conflict of Interest

The authors declare that the research was conducted in the absence of any commercial or financial relationships that could be construed as a potential conflict of interest.

## Publisher's Note

All claims expressed in this article are solely those of the authors and do not necessarily represent those of their affiliated organizations, or those of the publisher, the editors and the reviewers. Any product that may be evaluated in this article, or claim that may be made by its manufacturer, is not guaranteed or endorsed by the publisher.
